# Danshenol A Alleviates Hypertension-Induced Cardiac Remodeling by Ameliorating Mitochondrial Dysfunction and Suppressing Reactive Oxygen Species Production

**DOI:** 10.1155/2019/2580409

**Published:** 2019-09-11

**Authors:** Kai Chen, Yiqing Guan, Yunci Ma, Dongling Quan, Jingru Zhang, Shaoyu Wu, Xin Liu, Lin Lv, Guohua Zhang

**Affiliations:** ^1^School of Traditional Chinese Medicine, Southern Medical University, Guangzhou, China; ^2^Hong Kong University-Shenzhen Hospital, Shenzhen, China; ^3^Southern Medical University Nanfang Hospital, Guangzhou, China; ^4^School of Pharmaceutical Sciences, Southern Medical University, Guangzhou, China

## Abstract

Current therapeutic approaches have a limited effect on cardiac remodeling, which is characteristic of cardiac fibrosis and myocardial hypertrophy. In this study, we examined whether Danshenol A (DA), an active ingredient extracted from the traditional Chinese medicine *Radix Salviae*, can attenuate cardiac remodeling and clarified the underlying mechanisms. Using the spontaneously hypertensive rat (SHR) as a cardiac remodeling model, DA ameliorated blood pressure, cardiac injury, and myocardial collagen volume and improved cardiac function. Bioinformatics analysis revealed that DA might attenuate cardiac remodeling through modulating mitochondrial dysfunction and reactive oxygen species. DA repaired the structure/function of the mitochondria, alleviated oxidative stress in the myocardium, and restored apoptosis of cardiomyocytes induced by angiotensin II. Besides, DA inhibited mitochondrial redox signaling pathways in both the myocardium and cardiomyocytes. Thus, our study suggested that DA attenuates cardiac remodeling induced by hypertension through modulating mitochondrial dysfunction and reactive oxygen species.

## 1. Introduction

Cardiovascular disease is still a serious threat to senior citizens in aging countries [1]. Cardiac remodeling, caused by hypertension, coronary disease, valvulopathy, and other stimuli, takes part in the occurrence and development of various cardiovascular diseases and even results in chronic heart failure [2]. Unfortunately, an ideal solution for therapeutic cardiac remodeling is lacking. The pathophysiology of cardiac remodeling includes cardiomyocyte hypertrophy and apoptosis, as well as extracellular fibrosis, which are considered the major predictive indicators of mortality in sufferers of cardiovascular diseases [3, 4]. Therefore, researchers believed that cardiac function could be ameliorated by inhibiting cardiac remodeling.

Evidence has been increasing that oxidative stress serves as an important mechanism for myocardial remodeling and cardiac failure [5, 6]. As a matter of fact, oxidative stress occurs in all cardiovascular tissue and regulates a variety of cell functions such as cytodifferentiation, multiplication, caducity, and apoptosis under physiological conditions [7]. In cardiac remodeling, oxidative stress is always intensive, which is manifested as mitochondrial dysfunction and excessive generated reactive oxygen species (ROS). These effects ultimately lead to cardiac contractile failure and structural damage [8, 9]. Accordingly, targeting cardiac oxidative stress as a strategy to inhibit hypertensive cardiac remodeling has attracted considerable attention over the past decade.

Over the past decades, natural plant medicines have been extensively applied and popularized in numerous countries. The World Health Organization encourages the utilization of natural medicine as a promising adjuvant treatment strategy [10, 11]. *Radix Salviae* (Danshen) is a kind of traditional Chinese herb applied extensively in the treatment of cardiovascular diseases in China. As a drug for cardiovascular diseases, the compound Danshen dropping pill is currently undergoing phase III trials in clinical centers over nine countries. It is expected to become the first Chinese medicine authenticated by the Food and Drug Administration [12]. Danshenol A (DA) is an abietane-type diterpene ester separated from *Radix Salviae*. In contrast with the well-known tanshinone form of *Radix Salviae*, for instance, tanshinone I, tanshinone IIA, and cryptotanshinone, few studies have reported the biological effects of Danshenol. Previous reports indicated that the anti-inflammatory properties of DA are superior to those of tanshinone IIA, thereby prompting that DA has potential efficacy for atherosclerosis [13]. Another study revealed that DA can protect endothelial cells from oxidative stress by directly scavenging ROS [14]. Preliminary research conducted by our group found that treatment with DA significantly improves ventricular function in SHR and reduces ROS levels. The results aroused our interests. We surmise that the underlying mechanisms of DA attenuating cardiac remodeling are related to the oxidative stress pathway.

Our manuscript is intended at investigating the protective effects of DA on cardiac remodeling induced by hypertension and identifying whether the underlying mechanisms are associated with the oxidative stress pathway.

## 2. Materials and Methods

### 2.1. Reagents and Drugs

DA (CAS:189308-08-5, purity ≥ 98%) was purchased from EMMX Biotechnology LLC (Santiago, USA). Captopril tablets (12.5 mg) as the positive control were provided by Bristol-Myers Squibb Co. Ltd. (lot 1209031).

The biochemical kits for the detection of lactate dehydrogenase (LDH, batch number: A020-2), creatine kinase (CK, batch number: A032), CK-MB (batch number: H197), alanine aminotransferase (ALT, batch number: C009-2), aspartate aminotransferase (AST, batch number: C010-2), creatinine (Cr, batch number: C011-2), blood urea nitrogen (BUN, batch number: C013-2), and ROS (batch number: E004) were obtained from Nanjing Jiancheng Biotech Co. Ltd. (Nanjing, China). Biochemical kits of MitoCheck Complex I activity (lot 700930), MitoCheck Complex II activity assay kit (lot 700940), MitoCheck Complex II/III activity assay kit (lot 700950), and citrate synthase activity (lot 700990) were obtained from Cayman Chemical Company (Ann Arbor, Michigan).

### 2.2. Animal Grouping and Administration

Animal feeding and experimental procedures were conformed to institutional animal ethics committee guidelines, which acted in accordance with the Care and Use of Laboratory Animals published by the United States National Institutes of Health (NIH Publications No. 85-23, revised 1996).

Forty male spontaneously hypertensive rats (SHR) and eight male Wistar-Kyoto (WKY) rats at the age of 16 weeks were bought from Vital River Laboratory Animal Technology Co. Ltd. The rats were conventionally raised for 5 days in a SPF laboratory animal room at first, where the environment was set procedurally at 24°C ± 2°C and 35% ± 5% humidity under a regular 12 h/12 h light/dark schedule. All rats have free access to sterile water and forage.

Using a randomized complete control study, the forty SHR were assigned into five groups: SHR group (cardiac remodeling model, *n* = 8), CAP group (SHR+captopril, 13.5 mg·kg^−1^·day^−1^, *n* = 8), DAL group (SHR+DA low dose, 0.3 mg·kg^−1^·day^−1^, *n* = 8), DAM group (SHR+DA medium dose, 1 mg·kg^−1^·day^−1^, *n* = 8), and DAH group (SHR+DA high dose, 3 mg·kg^−1^·day^−1^, *n* = 8). The WKY group (blank control, *n* = 8) consisted of male Wistar-Kyoto rats. DA or captopril was orally administrated daily for 12 weeks, while the WKY and SHR groups received the equal volume of normal saline.

### 2.3. Blood Pressure and Echocardiography Measurements

After 12 weeks of administration, systolic blood pressure (SBP), diastolic blood pressure (DBP), and mean blood pressure (MBP) were determined using noninvasive tail pressure equipment (ALC-NIBP, Shanghai Alcott Biotechnology Co. Ltd., Shanghai, China). In brief, animals were preheated at 38°C for 8 minutes in a thermostat pad, and three stable consecutive measurements of blood pressure including SBP and DBP were recorded. Also, ejection fraction (EF) and fractional shortening (FS) were detected via color Doppler ultrasound diagnostic instrument in M-mode with a 10 MHz probe (S40 Exp).

### 2.4. Serum Sample Analysis

After treatment for 12 consecutive weeks, the blood samples were gathered from the caudal vein of rats and centrifugated at 3000 rpm/min for 12 min. Then, the serum was collected carefully from the supernatant and stored at −80°C. Myocardial injury was evaluated with the serum concentration of LDH, CK, and CK-MB, whereas liver and renal functions were examined using ALT, AST, Cr, and BUN.

### 2.5. Histopathological Detection

Executed with narcotic overdose, hearts from the rats were separated and weighed to determine the HW/BW index (the rate of heart weight to body weight). The left ventricle tissue was dissociated partially and fixed with 4% polyformaldehyde for 48 h. Then, the tissue was embedded in paraffin, sliced at 5 mm, and stained with Masson's trichrome (Solarbio, USA) to visualize fibrillar collagen. The extent of fibrosis was observed in 8 random fields of vision each sample and quantitated as the collagen volume fraction (CVF) using a light microscope (CX31, Olympus) at 40x magnification and ImageJ software (National Institutes of Health, Bethesda).

### 2.6. Analysis of Molecular Mechanisms

To clarify the molecular mechanisms of DA on cardiac remodeling, molecular targets of DA were obtained from the Traditional Chinese Medicine Systems Pharmacology Database and Analysis Platform (TCMSP, http://lsp.nwu.edu.cn/index.php) [15] and Bioinformatics Analysis Tool for Molecular mechANism of Traditional Chinese Medicine (BATMAN-TCM, http://bionet.ncpsb.org/batman-tcm) [16]. The targets were further screened using the PharmMapper server (http://lilab.ecust.edu.cn/pharmmapper/) [17]. Subsequently, we executed KEGG analysis by a plug-in ClueGO in Cytoscape 3.6.1 (https://cytoscape.org) [18] to show the signaling pathways related to DA on cardiac remodeling (*P* < 0.05, min overlap ≥ 3).

### 2.7. Electron Microscopy

Anterior walls of the left ventricle were transferred into 2.5% glutaraldehyde and 1% paraformaldehyde for 24 h. After washing at least twice with 0.1 M PBS at 4°C, 1% OsO_4_-buffered solution (pH 7.4) was used to postfix the tissue samples for 1 h. The resins were embedded and sectioned through the EM Ultramicrotome LKB-2088 and stained using 1% toluidine blue solution. The ultrathin sections were then stained with uranyl acetate and lead citrate twice. Eventually, the morphology of mitochondria in the myocardium was observed using an electron microscope (Hitachi H-7500).

### 2.8. Mitochondrial Complex Activity

Mitochondria in heart tissue were extracted as described above, and the activity of the mitochondrial complex was examined by corresponding biochemical kits. In brief, complex I activity was measured as the oxidation extent of nicotinamide adenine dinucleotide. Complex II activity was measured as the diminution in artificial electron acceptor L2 6-dichlorophenolindophenol. Complex III activity was determined as the reduction of cytochrome c. Complex IV was determined by the reduction in acetyl-CoA, which was measured as the content of oxaloacetate.

### 2.9. Adenine Nucleotide Analysis

The heart tissue and cardiomyocytes were collected and immersed in 0.6 M HClO_4_ (4 mL/g, 4°C), then homogenized directly and transferred into a centrifuge at 10000 r/min for 15 min. The supernatant was neutralized and filtered after centrifugation under the same conditions. The test solution was completely separated with isocratic elution via 96% 0.05 M KH_2_PO_4_ (pH 6.5) and 4% methanol for 20 min in high-performance liquid chromatography (HPLC) with a Waters C18 column (250 × 4.6 mm, 5 *μ*m). Content of adenosine triphosphate (ATP), adenosine diphosphate (ADP), and adenosine monophosphate (AMP) was measured at 254 nm via an external standard method for quantification. The energy charge was calculated as ((ATP + ADP)/2)/(ATP + ADP + AMP) [19].

### 2.10. Determination of Oxidative Stress in the Myocardium

After treatment for 12 consecutive weeks, myocardium samples were collected as mentioned above. The level of oxidative stress was determined by measuring the concentrations of ROS, malondialdehyde (MDA), and 4-hydroxynonenal (4-HNE) in the myocardium of rats.

### 2.11. Cell Culture

Primary cultures of rat cardiomyocytes were conducted in accordance with previous studies. In brief, neonatal Sprague-Dawley rats were sacrificed. The hearts were rapidly separated from the rats and washed using phosphate-buffered saline. Then, cardiac tissues were minced via amicrobic scissors and digested using Hanks' solution with 0.1% trypsin at 37°C for 3 min. Cells were isolated by digestion for 8–10 times and transferred to Dulbecco's modified Eagle medium containing 10% fetal bovine serum (FBS). The cardiomyocytes were then cultivated in culture flasks with a density of 1 × 10^5^ cells/cm^2^ and plated into an incubator where the environment was maintained at 37°C with 5% CO_2_.

### 2.12. Detection of Apoptosis

The apoptosis rate of cardiomyocytes was determined via an Annexin V-FITC Apoptosis Detection Kit and a flow cytometer. Cardiomyocytes were cultivated in six-well culture plates with Dulbecco's modified Eagle medium with 10% FBS for 48 h. The medium was replaced with serum-free medium and cultivated for 24 h. Then, cells were pretreated with DA (1, 3, and 10 *μ*mol/L) and captopril (5 *μ*mol/L) for 35 min, followed by 0.1 *μ*mol/L angiotensin II (Ang II) for another 48 h. After incubation, cells were cleaned with PBS and resuspended. Then, fluorescein-conjugated annexin V (5 mL) and propidium iodide reagent (5 mL) were added to cell suspensions. The mixture above was incubated for 15 min protected from light. Finally, the rate of apoptosis was determined and quantified by FACScan flow cytometry (Beckman Coulter).

### 2.13. Western Blot

The total proteins were isolated from the myocardial tissue and cardiomyocytes. After quantitation, protein samples were analyzed using 15% gradient gel and then transferred into the polyvinylidene fluoride (PVDF) membrane via a gel transfer device. Then, PVDF membranes were incubated via primary antibodies against Bax, Bcl-2, (GTP)p-Ras, Ras, p-Raf, Raf, p-Mek, Mek, p-Erk, Erk, Ask1, p-Jnk, Jnk, p-p38, p38, and GAPDH overnight at 4°C. After that, the primary antibodies were cleaned with Tween20/TBS solution, and then secondary antibodies were incubated for another 1 h. Using developing solution, the expression of proteins was measured via an Odyssey infrared imaging system (LI-COR Biosciences) and normalized to the GAPDH protein level.

### 2.14. Statistical Analysis

Results are presented as the mean ± standard deviation. Statistical analysis was conducted using one-way ANOVA and Dunnett's test. *P* value less than 0.05 was defined by having the difference of statistics. Statistical analysis was determined via GraphPad Prism 5.01 for Windows.

## 3. Results

### 3.1. Effect of DA on Blood Pressure and Cardiac Function

**Figure 1:**
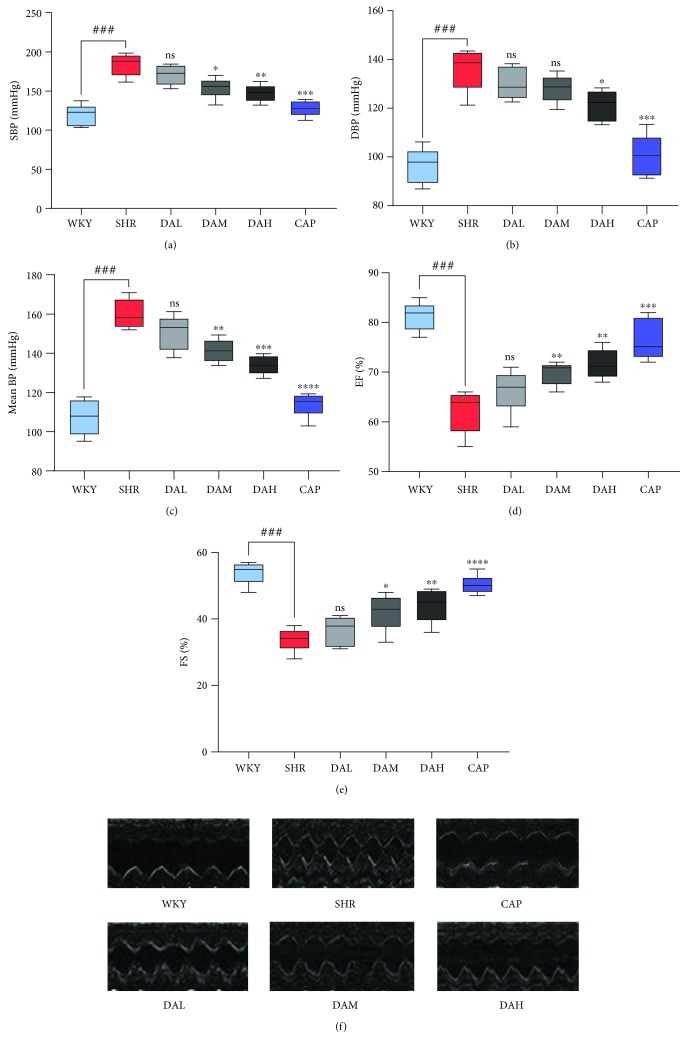
Effects of DA treatment on blood pressure and cardiac function during administration for 12 consecutive weeks: (a) SBP; (b) DBP; (c) MBP; (d) EF; (e) FS; (f) image of ultrasound cardiogram. *n* = 7. ^###^*P* < 0.001 vs. WKY, ^##^*P* < 0.01 vs. WKY, and ^#^*P* < 0.05 vs. WKY; ^∗∗∗^*P* < 0.001 vs. SHR, ^∗∗^*P* < 0.01 vs. SHR, and ^∗^*P* < 0.05 vs. SHR.

Dose-related alternations in SBP, DBP, and MBP for the six groups are exhibited in Figures 1(a) and 1(b). After treatment for 12 consecutive weeks, SBP, DBP, and MBP in the SHR group were obviously higher than those in the WKY group (Figure 1(a)–1(c)). With DA administration, blood pressure was decreased, which showed a dose-dependent manner, but the overall effects were not as credible as those in the CAP group.

EF and FS were used to evaluate the impairment of cardiac function, and the impairment of EF and FS suggested cardiac functional insufficiency. As shown in Figures 1(d) and 1(e), EF and FS in SHR were substantially deficient than those in WKY, whereas DA and CAP treatments increased EF and FS. Representative ECG images in M-mode are shown in Figure 1(f).

### 3.2. Effects of DA Treatment on Cardiac, Hepatic, and Renal Functions

**Figure 2:**
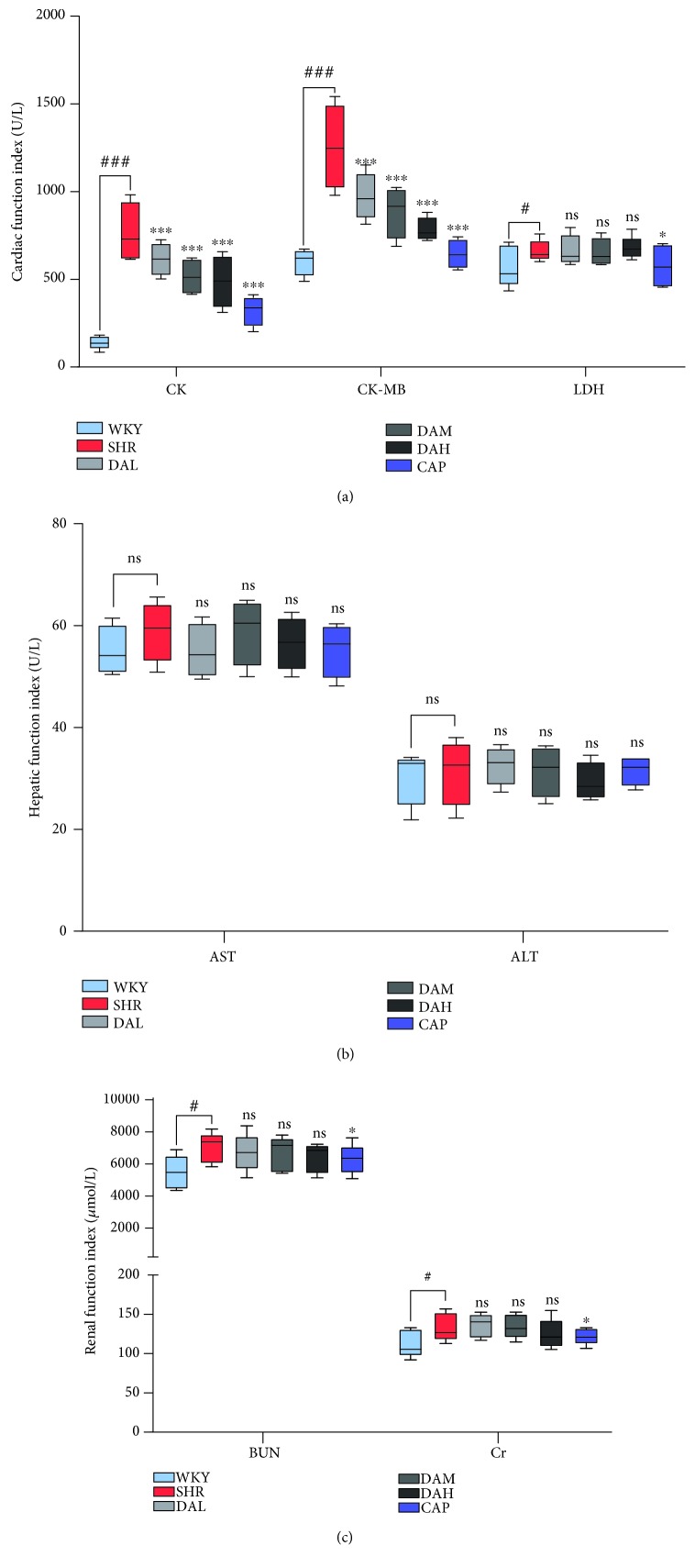
Effect of DA treatment on the serum biochemical index at 12 weeks. (a) Cardiac function index including CK, CK-MB, and LDH; (b) hepatic function index including AST and ALT; (c) renal function index including Cr and BUN. ^###^*P* < 0.001 vs. WKY, ^##^*P* < 0.01 vs. WKY, and ^#^*P* < 0.05 vs. WKY; ^∗∗∗^*P* < 0.001 vs. SHR, ^∗∗^*P* < 0.01 vs. SHR, and ^∗^*P* < 0.05 vs. SHR.

To estimate the protection of DA on the heart and its safety for the liver and kidney, indexes of a myocardial enzymogram (CK, CK-MB, and LDH), liver function (ALT, AST), and kidney function (Cr, BUN) were detected in the present study. As shown in Figure 2, the serum concentration of CK and CK-MB increased obviously in SHR compared with WKY (*P* < 0.001), indicating that hypertension induced heart damage (Figure 2(a)). With treatment, DA improved cardiac function as evidenced by the reduction in serum CK and CK-MB, whereas the CAP group was the most effective group.

### 3.3. Effects of DA Treatment on Cardiac Remodeling

**Figure 3:**
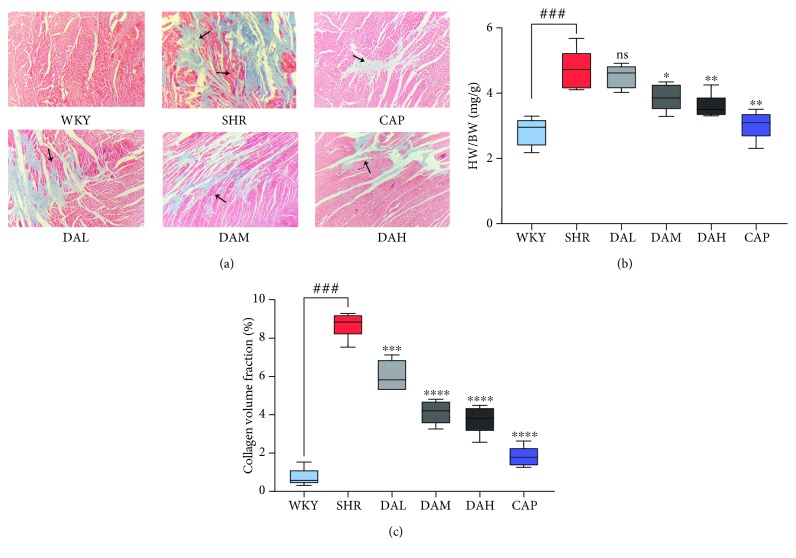
Effects of KXF treatment on cardiac remodeling. (a) Masson staining images under 200x magnification and the areas of collagen deposition were indicated by the black arrow; (b) HW/BW; (c) CVF. Results are presented as the mean ± SEM (^∗^*P* < 0.05, ^∗∗^*P* < 0.01, and ^∗∗∗^*P* < 0.001 vs. SHR; ^###^*P* < 0.001 vs. WKY).

Consistent with the alternations in blood pressure and cardiac function, significant deterioration of cardiac remodeling under histological analysis was determined in the SHR compared with the WKY group (Figure 3(a)), as evidenced by the increase in the ratio of HW/BW (Figure 3(c)). The treatment of DA prevented cardiac remodeling and decreased HW/BW compared with SHR.

Masson's trichrome-stained sections (Figure 3(b)) and CVF (Figure 3(d)) showed that the content of cardiac collagen (staining in blue color) under DA treatment was obviously relieved compared with the SHR group (40x magnification), which showed a dose-dependent manner.

### 3.4. Molecular Mechanisms of DA on Cardiac Remodeling

**Figure 4:**
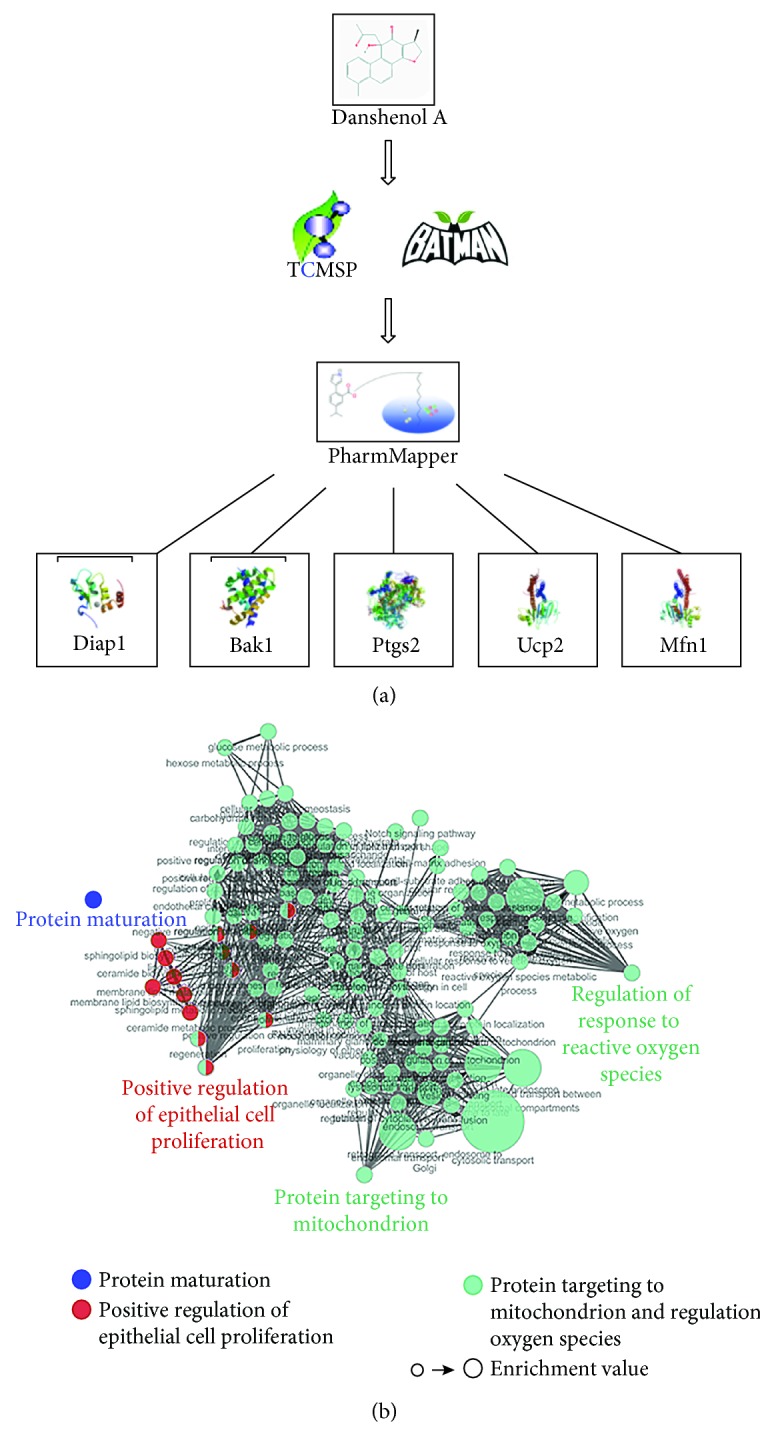
KEGG analysis of signaling pathways related to DA on cardiac remodeling. (a) Flow chart of reverse molecular docking using PharmMapper. (b) Significant enrichment of protein targets in the network diagram.

Using the TCMSP database and BATMAN-TCM, we obtained 25 potential molecular targets of DA (Table 1). Molecular docking was carried out to increase the reliability of the results. Twelve molecular targets, namely, Ptgs1, Kcnc2, F10, Ptgs2, Diap1, Daf-2, Pik3cg, Mfn1, Ace, Cox17, Arnt, and Prkca, were screened as molecular targets of DA (Table 2). Signaling pathways enriched from the molecular targets are shown in Figure 4 and Table 3. Consequently, protein targeting to the mitochondrion and regulation of the response to ROS may be the potential signaling pathway underlying DA treatment of cardiac remodeling.

### 3.5. Effect of DA on Mitochondrial Morphology and Mitochondrial Complex Activity

**Figure 5:**
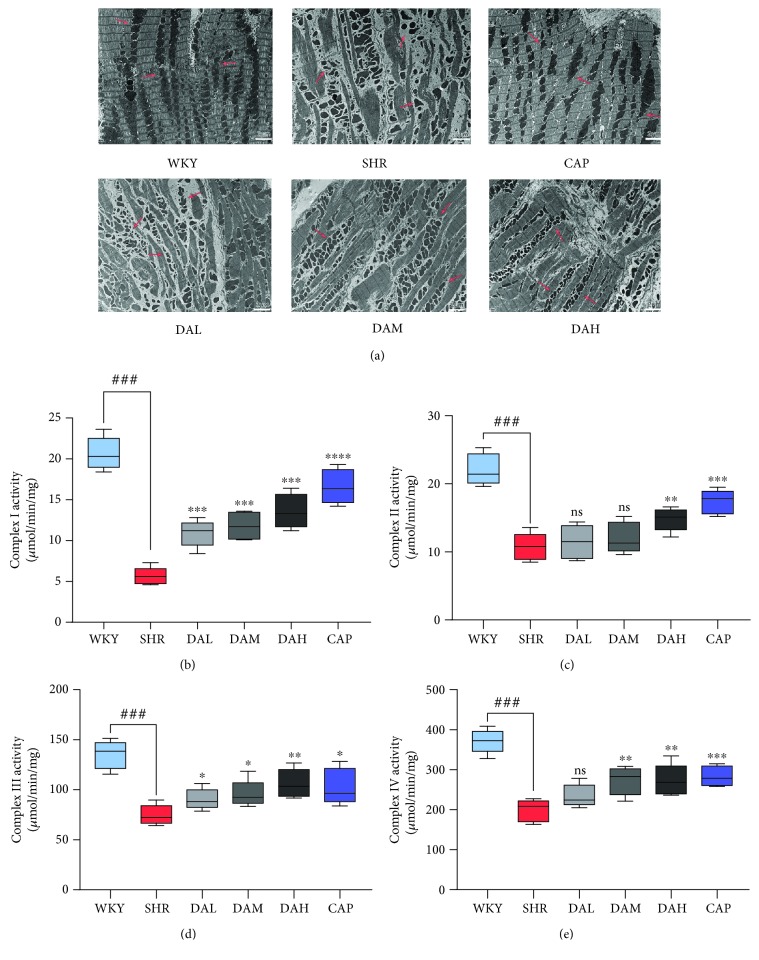
Changes in mitochondrial morphology and mitochondrial complex activity. (a) Ultrastructural analysis of myocardium under 8000x magnification and the areas of the mitochondrion were marked by the black arrow. (b–e) Maximal activities of complexes I-IV in cardiac mitochondria. ^###^*P* < 0.001 vs. WKY, ^##^*P* < 0.01 vs. WKY, and ^#^*P* < 0.05 vs. WKY; ^∗∗∗^*P* < 0.001 vs. SHR, ^∗∗^*P* < 0.01 vs. SHR, and ^∗^*P* < 0.05 vs. SHR.

Electron microscopy observations on the cardium showed obvious morphological changes in the SHR group compared with the WKY group (Figure 5(a)). The density of the mitochondrium was obviously decreased in the cardium of the SHR group compared with the WKY group. Furthermore, the structure of the cardium in the SHR group showed defective striation, reduced cristae structures, and disappearance of the *z* line compared with that in the WKY group. By contrast, DA treatment restored the damage to mitochondrial morphology and cardium structure.

Maximal activities of complexes I–VI were determined in cardiac mitochondria to evaluate the function of cardiac mitochondria. Mitochondrial complex enzyme activities decreased in the SHR group than in the WKY group, whereas DA treatment enhanced the activities, which showed a dose-dependent manner. (Figure 5(b)–5(e)).

### 3.6. Effect of DA on Mitochondrial Function and Oxidative Stress

**Figure 6:**
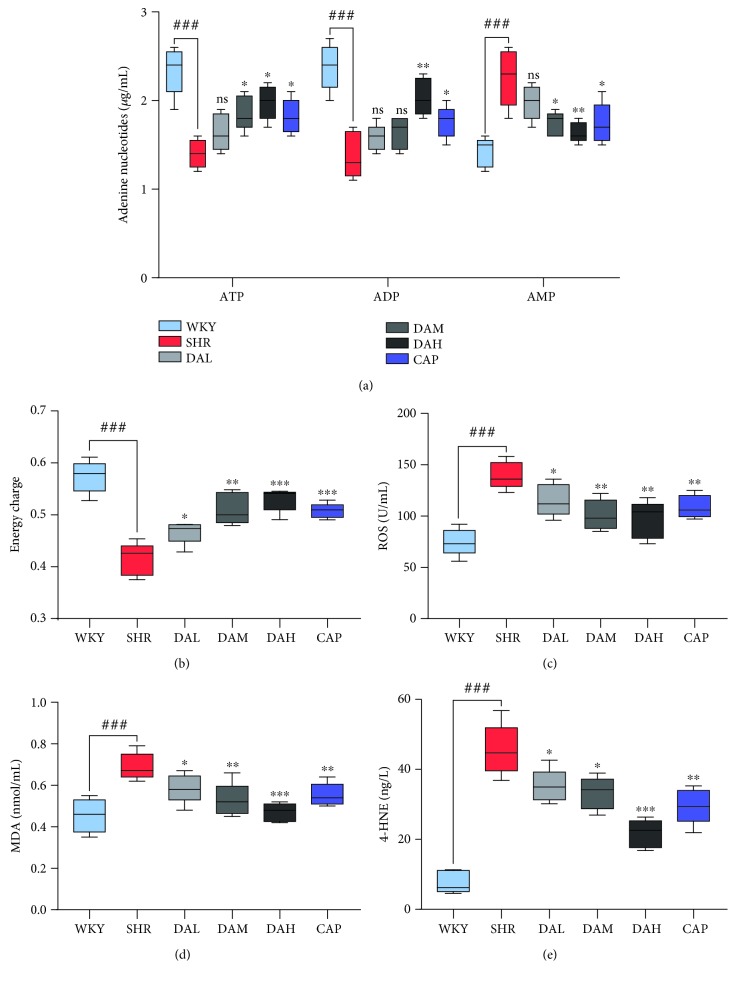
(a, b) ATP, ADP, and AMP concentrations and energy charge in the myocardium. (c–e) Oxidative stress indexes of MDA, ROS, and 4-HNE concentrations in the myocardium. ^###^*P* < 0.001 vs. WKY, ^##^*P* < 0.01 vs. WKY, and ^#^*P* < 0.05 vs. WKY; ^∗∗∗^*P* < 0.001 vs. SHR, ^∗∗^*P* < 0.01 vs. SHR, and ^∗^*P* < 0.05 vs. SHR.

The measurement of adenine nucleotide variants was conducted to evaluate the energy production in myocardial mitochondria. As a result, DA treatments increased ATP and ADP content in the myocardium, while ATP and ADP content in the SHR group was obviously lower than that in the WKY group (Figure 6(a)). Besides, the AMP content was more abundant in the SHR group compared with the WKY group, whereas DA treatment reduced it. Overall, DA groups, especially the high dose groups, demonstrated higher energy charge compared with the SHR group (Figure 6(b)). Consequently, the expression of ROS, MDA, and 4-HNE obviously increased in the SHR group (Figure 6(c)–6(e)), but the upregulated expression was less pronounced in the DA groups.

### 3.7. Effect of DA on Cardiomyocyte Apoptosis

**Figure 7:**
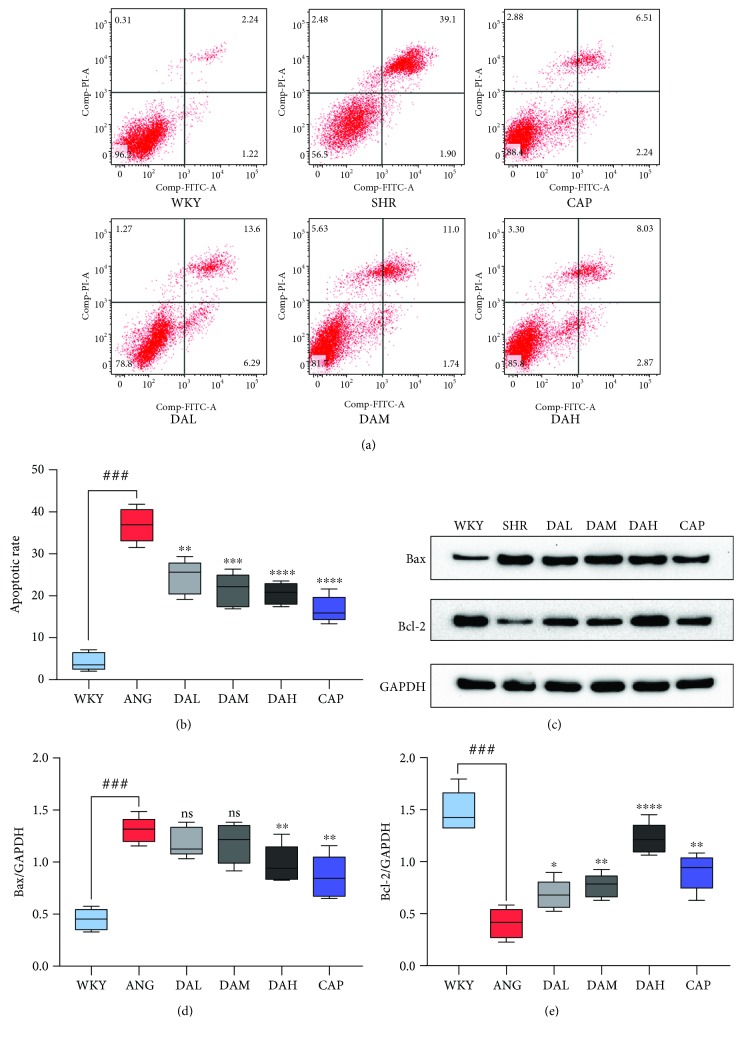
Effect of DA on cardiomyocyte apoptosis. (a, b) Ang II-induced cell apoptosis rate was quantified by flow cytometry. (c–e) Western blot analysis of apoptosis-related proteins: Bcl-2 and Bax in each groups. ^###^*P* < 0.001 vs. WKY, ^##^*P* < 0.01 vs. WKY, and ^#^*P* < 0.05 vs. WKY; ^∗∗∗^*P* < 0.001 vs. SHR, ^∗∗^*P* < 0.01 vs. SHR, and ^∗^*P* < 0.05 vs. SHR.

Incubation with Ang II (0.1 *μ*mol/L) for 48 h enhanced the apoptotic rate of cardiomyocytes (Figures 7(a) and 7(b)) with increased expression of apoptotic protein Bax and decreased expression of apoptosis inhibitory protein Bcl-2. By contrast, pretreatment with DA (1, 3, and 10 *μ*mol/L) obviously decreased the apoptotic rate of cardiomyocytes incubated by Ang II, which was accompanied with the decreased expression of Bax and increased expression of Bcl-2 (Figure 7(c)–7(e)).

### 3.8. Effect of DA on Mitochondrial Redox Signaling Pathways

**Figure 8:**
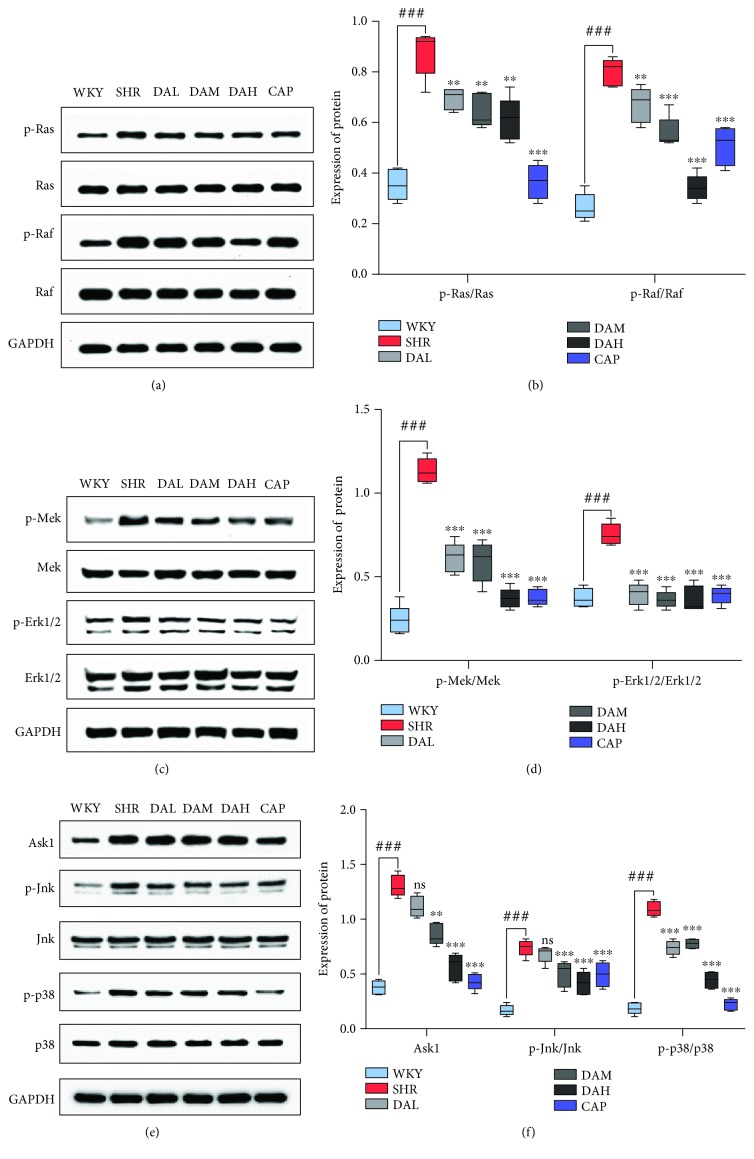
Mechanism of DA ameliorates cardiac remodeling involved in mitochondrial redox signaling pathways in the myocardium. Protein expression of (a, b) p-Ras, Ras, p-Raf, and Raf; (c, d) p-Mek, Mek, p-Erk, and Erk; and (e, f) Ask1, p-Jnk, Jnk, p-p38, and p38 in the myocardium. Results are presented as the mean ± SEM (^∗^*P* < 0.05, ^∗∗^*P* < 0.01, and ^∗∗∗^*P* < 0.001 vs. SHR; ^∗^*P* < 0.05, ^∗∗^*P* < 0.01, and ^∗∗∗^*P* < 0.001 vs. Ang II; ^###^*P* < 0.001 vs. WKY and CON).

**Figure 9:**
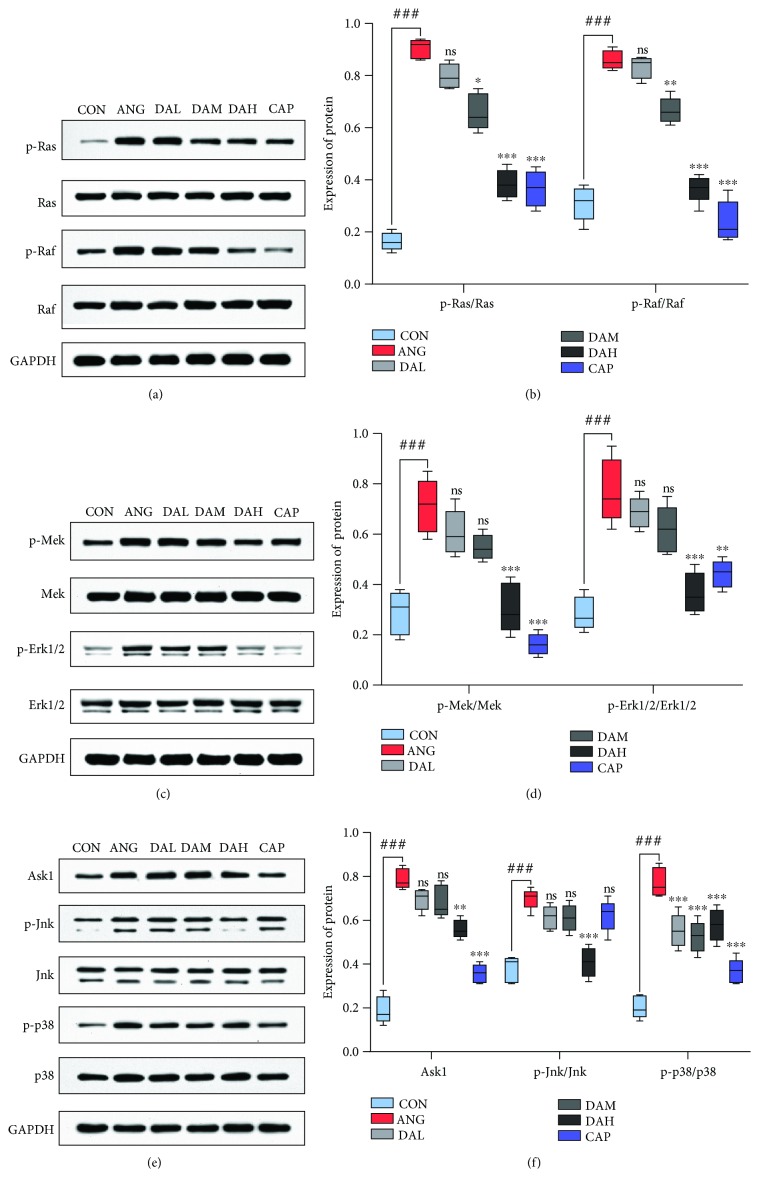
Mechanism of DA ameliorates cardiac remodeling involved in mitochondrial redox signaling pathways in cardiomyocytes. Protein expression of (a, b) p-Ras, Ras, p-Raf, and Raf; (c, d) p-Mek, Mek, p-Erk, and Erk; and (e, f) Ask1, p-Jnk, Jnk, p-p38, and p38 in cardiomyocytes. Results are presented as the mean ± SEM (^∗^*P* < 0.05, ^∗∗^*P* < 0.01, and ^∗∗∗^*P* < 0.001 vs. SHR; ^∗^*P* < 0.05, ^∗∗^*P* < 0.01, and ^∗∗∗^*P* < 0.001 vs. Ang II; ^###^*P* < 0.001 vs. WKY and CON).

Mitochondrial redox signaling pathways are mainly regulated by Ras, Raf, Mek, Erk, Ask1, Jnk, and p38. The result of Western blot showed that the protein levels of p-Ras, p-Raf, p-Mek, p-Erk, Ask1, p-Jnk, and p-p38 were raised in the SHR group than in the WKY group, while pretreatment with DA decreased those (Figure 8). A similar result was observed in cardiomyocytes (Figure 9).

## 4. Discussion

Cardiac remodeling, an adaptive response of the heart to pressure overload, is a result from physiological or pathological stimuli [20], which presented as abnormal thickening of the ventricular wall and reduced volume of the ventricular chamber [21]. Although the physiological processes maintain enhanced heart function, cardiac remodeling can be decompensated and deteriorated into heart failure under pathological conditions [22]. Pathological hypertrophy is accompanied by the high fetal gene expression, excessive fiber deposition, and cardiac dysfunction. Research also suggested that the pathogenesis of cardiac remodeling is related to oxidative stress [6]. We used the SHR models to illustrate the ameliorated cardiac dysfunction effect of DA and its antioxidant capacity in vivo.

SHR is a mature hypertensive model that was introduced in 1963, in which myocardial damage occurs and eventually leads to cardiac remodeling after 16 weeks [23]. Hence, SHR was used as a cardiac remodeling model in this study. Captopril is an angiotensin-converting enzyme inhibitor that is widely used in the treatment of hypertension and congestive heart failure. Captopril was utilized as a positive control due to its ability to reverse cardiac fibrosis function.

The present study indicated that DA treatment significantly decreased SBP and DBP in SHR, but the effects were inferior to captopril. EF and FS are the fractions of outbound blood pumped from the heart in each cardiac cycle. EF and FS, which are measured by an echocardiogram, are general indicators of cardiac function [24]. As a result, EF and FS were enhanced in DA-treated groups compared with the SHR group, thereby illustrating that DA ameliorated cardiac function. Numerous cytokines have been demonstrated to be bound up with cardiac damage and cardiac dysfunction. Coinciding with previous studies, our data showed that the plasma content of CK, CK-MB, and LDH in the DA-administrated group was reduced compared with that in the SHR group. Meanwhile, Masson staining demonstrated a decrease in CVF in the DA-administrated group relative to the SHR group. Thus, DA treatment decreased cardiac injury markers of function in SHR, repaired the injured myocardium, and ameliorated cardiac function.

To explore the specific mechanism of DA on cardiac remodeling, bioinformatics technology was used in this study. TCMSP is an advanced platform of network pharmacology for Chinese herbal medicine which contains the correlations among drugs, targets, and diseases [15]. BATMAN-TCM, which captures TCM-related data obtained from different platforms, constructs a network model for integrative relationships among herbs, ingredients, targets, and diseases [16]. PharmMapper server conducts an in silico target prediction algorithm for a given small molecule through “probing” of the potential ligand binding sites via pharmacophore models [17]. Using these databases, we obtained potential molecular targets of DA. Functional enrichment analysis was carried out to show the mechanism of DA based on the targets, which, using a hypergeometric test to identify the significant enrichment pathway in differential proteins compared with all identified proteins, can identify the main biochemical metabolic pathways involved.

Accordingly, the analysis determined that the molecular targets were significantly enriched in functions associated with “protein targeting to mitochondrion/regulation of response to reactive oxygen species,” “positive regulation of epithelial cell proliferation,” “protein maturation,” and “amoebiasis.” Among them, “protein targeting to mitochondrion/regulation of response to reactive oxygen species” was highly enriched with a target level of 6 and considered one of the most essential pathways modulating the mechanism of DA. On the basis of the annotation data of gene ontology [25], the definition of “protein targeting to mitochondrion” is the process that modulates the frequency, rate, or extent of protein targeting to the mitochondrion, whereas “regulation of response to reactive oxygen species” is the process that modulates the frequency, rate, or extent of response to ROS. The annotation of enrichment is quite a general concept, which only serves as a hint, so we focus on the relationship between the mitochondrion, ROS, cardiac remodeling, and DA through previous studies.

Growing evidence indicates that ROS is essential for the pathogenesis of cardiac remodeling, which may also exert an obvious effect in the progression from pathological remodeling to heart failure [26, 27]. For example, Dirican et al. reported a negative relevant relation between MDA and LVEF [28]. Yokota et al. found that the activation of SOD, catalase, and GSHIPx did not decrease in failing hearts, which indicated that oxidative stress in heart failure is mainly owing to the increase of prooxidant generation rather than to the reduction of antioxidant defenses [29]. Excess ROS can also attack the components of the mitochondria, which results in mitochondrial dysfunction and oxidative damage, and ultimately initiate cell death such as apoptosis [30]. Given the highest oxygen uptake rate of the heart in the body, cardiomyocytes have the highest volume density of mitochondria, accounting for 40%–60% of the total volume of cardiomyocytes [31]. Under compensatory effect, a small amount of ROS is generated from mitochondrial respiration and detoxification can be achieved through the endogenous scavenging mechanisms of cardiomyocytes [32]. Nevertheless, mitochondrial dysfunction will lead to the chronic release of ROS under decompensation. This toxicity accumulation can eventually cause cardiac remodeling and progression of heart failure [33]. During the early stages of pathological hypertrophy, mitochondrial failure is related to the decrease in complex enzyme activity and impairment of mitochondrial ATP generation [34]. Therefore, the mitochondria are a primary source of ROS in heart failure, which also indicates a pathophysiological relationship between mitochondrial dysfunction and oxidative stress. Our results indicated that mitochondria were also damaged in the case of myocardial injury, showing increased permeability of mitochondrial membranes (Figure 5); decreased activity of respiratory chain complexes I, II, III, and IV; and decreased energy metabolism and total energy charge in the SHR group compared with the WKY group. Compared with WKY, the ROS, MDA, and 4-HNE levels were significantly decreased in SHR. After treatment with DA for 3 months, the above indicators were all reversed.

Mitochondrial dysfunction will amplify apoptotic signals, and cardiomyocyte apoptosis plays a crucial role in cardiac remodeling and heart failure [35, 36]. Previous studies showed that Ang II could induce mitochondrial dysfunction, which presented with increased permeability of mitochondrial membranes [37]. In the present study, Ang II evidently increased the rate of apoptotic cells in cardiomyocytes accompanied by mitochondrial dysfunction, whereas DA treatments decreased the apoptosis ratio and enhanced mitochondrial dynamics.

Extensive reports implicated that ROS generated from mitochondrial dysfunction can result in the release of remodeling factors including Raf, Ras, Mek, Erk1/2, Ask1, JNK, and p38. Ask1 is intensively provoked by ROS, and it then activates MAPKs p38 and JNK. The deletion of Ask1 decreases p38 and JNK activation and improves cardiac remodeling induced by Ang II, which were validated in our results. Downstream signaling pathways mediated by ROS were activated under the pathological conditions of ventricular remodeling in vivo and in vitro. The downstream signaling pathways included the MAPK classical pathway, ASK1/JNK pathway, and p38 MAPK pathway, and the expression of proteins in those pathways significantly increased. All of these responses were significantly reversed by DA treatment in primary cardiomyocytes and in the myocardium of SHRs. Thus, DA may improve cardiac remodeling by modulating mitochondrial redox signaling pathways.

## 5. Conclusion

In summary, our study demonstrated that DA could improve cardiac function, ameliorate cardiac fibrosis, restore mitochondrial structure/function in SHR, and decrease the apoptosis of cardiomyocytes. These functions may be completed by promoting mitochondrial dysfunction and reducing reactive oxygen species. We also proved that the mechanism may involve inhabitation of mitochondrial redox signaling pathways in cardiomyocytes and myocardium.

## Figures and Tables

**Table 1:** Targets of DA from TCMSP and BATMAN-TCM.

TCMSP	BATMAN-TCM
Ptgs1, Kcnc2, Scn5a, F10, Ptgs2, Rxra, Pik3cg, Ncoa1, and Kcnma1	Diap1, Bak1, Ptgs2, Ucp2, Mfn1, Mfn2, Mapl, Rxra, Ace, Pde3a, Daf-2, Adra1b, Aif, Hif1a, Arnt, Prkca, Cox17, Eif4e

**Table 2:** Targets of DA validated by PharmMapper server.

PharmMapper server
Ptgs1, Kcnc2, F10, Ptgs2, Diap1, Daf-2, Pik3cg, Mfn1, Ace, Cox17, Arnt, Prkca

**Table 3:** Enrichment analysis of protein targets related to DA.

Function	Count	FDR
Protein targeting to mitochondrion/regulation of response to reactive oxygen species	6	0.002487
Positive regulation of epithelial cell proliferation	4	0.042185
Protein maturation	4	0.041617
Amoebiasis	3	0.026515
AGE-RAGE signaling pathway	3	0.031415
MAPK signaling pathway	3	0.003792
HIF-1 signaling pathway	3	0.019821
EGFR tyrosine kinase inhibitor resistance	2	0.032885

## Data Availability

The data used to support the findings of this study are available from the corresponding authors upon request.

## Abbreviations

DA: Danshenol A
SHR: Spontaneously hypertensive rat
WKY: Wistar-Kyoto
SBP: Systolic blood pressure
DBP: Diastolic blood pressure
MBP: Mean blood pressure
EF: Ejection fraction
FS: Fractional shortening
CK: Creatine kinase
CK-MB: Creatine kinase-MB
ALT: Alanine aminotransferase
AST: Aspartate aminotransferase
Cr: Creatinine
BUN: Blood urea nitrogen
ROS: Reactive oxygen species
4-HNE: 4-Hydroxynonenal
CVF: Collagen volume fraction
HPLC: High-performance liquid chromatography.
